# Is Social Media Use a Blessing or Cure for Motor Function and Skill Acquisition? An Opinion Paper

**DOI:** 10.1523/ENEURO.0382-25.2026

**Published:** 2026-03-05

**Authors:** Lina Fricke, Thomas Wendeborn, Patrick Ragert

**Affiliations:** ^1^Departments of Movement Neuroscience, Leipzig University, Leipzig 04109, Germany; ^2^Sports Pedagogy, Faculty of Sport Science, Leipzig University, Leipzig 04109, Germany; ^3^Department of Neurology, Max Planck Institute for Human Cognitive and Brain Sciences, Leipzig 04103, Germany

**Keywords:** action observation, cognitive function, functional plasticity, motor skill learning, social media, structural plasticity

## Significance Statement

Social media (SM) use is typically regarded as a technological tool which might negatively impact physical fitness and cognitive function, especially in critical developmental stages. In this opinion paper, we argue that specific forms of SM content might be beneficial for promoting motor skill acquisition and function. Furthermore, we suggest that SM use might be a promising innovative tool for educational purposes in optimizing skills not only in sports but also in academia. As a prerequisite, future research is needed to clarify the optimal type of content, its use, and how these parameters are affected by age. Therefore, longitudinal studies across the lifespan are necessary for a thorough understanding of the potential beneficial effects of SM use.

## Introduction

The use of social media (SM) has rapidly accelerated due to technological progress and the release of the first SM platforms such as Facebook in the early 2000s. During the Covid-19 pandemic, lockdowns and social distancing acted as catalysts, contributing to the omnipresence of SM in our society over the recent years (González-Padilla and Tortolero-Blanco, 2020). In particular, the number of SM users has doubled over the last decade, rising from 2.27 billion in 2015 to 5.66 billion in 2025 (Kemp, 2025). In parallel to this development, the time spent on SM platforms increased continuously by ∼1 h between 2012 and 2018, reaching an average of 2.2 h. Since 2018, the global SM usage duration has plateaued at this level with slight increases during the Covid-19 pandemic (Thuy, 2025). Although SM usage duration decreases progressively with age in adulthood, it is not restricted to younger individuals but is ubiquitous across all age groups. Additionally, gender-specific differences have also been reported where generally women show higher SM usage as compared with men (Kemp, 2025).

SM are a group of internet-based applications that allow the creation, exchange, and aggregation of user-generated content (Davis, 2016). However, SM is a broad umbrella term since SM platforms differ in their complexity, features, and content and provide unique/platform-specific user experience (Parry et al., 2022). Commonly, social networking sites such as Facebook, TikTok, or Instagram are seen as SM platforms, as they provide social interaction and user-generated content. In addition, messenger applications like WhatsApp or Telegram are often classified as SM platforms as well.

However, SM and its distributing platforms are constantly changing their usage characteristics. Over the last two decades, SM platforms have transformed from simple communication tools to algorithmic-driven smartphone applications that are perfectly aligned with the individual user preferences. In addition, the type of content has shifted from predominantly text-based to primarily photo- and video-based formats (Kullolli and Trebicka, 2023).

Recent research on SM use provided compelling evidence that it negatively affects various behavioral domains including physical and mental health and is accompanied by alterations in brain function and structure (Korte, 2020). Excessive SM use has been shown to be associated with behavioral and neural alterations similar to those observed in other forms of addiction (Wadsley and Ihssen, 2023). Furthermore, excessive SM use may affect physical health since it is typically associated with an increased sedentary behavior (Moreno-Llamas et al., 2020) which in turn is related to a higher BMI (Martínez-González et al., 1999) and lower levels of physical fitness such as reduced muscular strength, cardiorespiratory fitness, and balance abilities (Silva et al., 2020).

Such consequences of SM use are concerning since maintenance of physical activity is crucial in all stages of life (Strain et al., 2024). In childhood, for example, physical activity is known to be important for the development of motor competence (Robinson et al., 2015). Even in older adults, higher fitness levels and an active lifestyle seem to be associated with successful aging. This in turn may delay and/or attenuate the age-related decline in motor and cognitive function (Hübner and Voelcker-Rehage, 2017).

Until now, most studies have focused on the (negative) effects of SM use on cognitive processes. While the consequences of SM use on mental health and cognitive functioning are well investigated (Huang, 2022; Shannon et al., 2022), the specific influence of SM use on motor functions and skill acquisition still remains elusive. This gap in research is particularly important given the growing concerns about the global rise in physical inactivity (Strain et al., 2024).

## SM Use and Its Effects on Cognitive and Motor Function

SM use has been repeatedly associated with negative effects on a huge variety of cognitive functions such as mental fatigue, changes in emotional states, memory performance, and attentional regulation (Korte, 2020).

The psychological impact of SM use seems to depend on how users engage with the respective SM platforms. Excessive SM use has been shown to negatively affect self-esteem, depressive symptoms, anxiety, and loneliness (O’Day and Heimberg, 2021). In addition to emotional processing, SM use might also affect memory performance. For example, Spence et al. (2020) showed that short-term memory performance was impaired when participants received new auditory information while simultaneously using Instagram.

Furthermore, the impact of SM use on cognitive processes might depend on the platform-specific character. Short-video content, embedded in popular SM platforms such as TikTok, Instagram, and YouTube, may act as a primary driver that impairs cognitive functioning. Reasons may be the rapidly shifting character of such content, driven by algorithmic designs that are specifically optimized to maximize user engagement and screen time (Goldon, 2024).

This phenomenon can be understood within the framework of SM fatigue, a cognitive state in which users are overwhelmed by the volume of information they consume. Consequently, users could feel mentally exhausted and may be unable to fully process respective content (Ravindran et al., 2014). Hence, daily exposure to short-video content seems to be negatively associated with working memory, verbal abilities, and overall academic performance in adolescents (Xu et al., 2023).

Nevertheless, as SM use is a relatively new phenomenon in our society, its long-term impact on cognitive function is still not well understood and needs to be further investigated, especially over the lifespan. In fact, excessive SM use during critical developmental periods has been assumed to impair cognitive function which in turn may manifest in neurodegenerative disease in later life (Manwell et al., 2022).

Apart from altered cognitive functions associated with SM use, it is reasonable to speculate that motor functions are negatively affected as well since (excessive) SM use has been associated with increased sedentary behavior (Moreno-Llamas et al., 2020).

In fact, short-term SM use prior to physical activity has been shown to impair visuomotor performance. Fortes et al. (2022) demonstrated that repeated 30 min Instagram use prior to volleyball training sessions (five to six times per week for two weeks) negatively impacts elite volleyballers’ performance in a sport-unspecific visuomotor reaction time task. Interestingly, the decline in visuomotor performance seems to be specific for SM use since watching a documentary showed opposite effects. Additionally, SM use might negatively impact resistance training performance, particularly by decreasing movement velocity in a bench press task (Alix-Fages et al., 2023).

On the other hand, Faro et al. (2025) found that SM use prior to a sport-specific visuomotor task did not affect motor performance. Furthermore, while short-term SM use between practice trials negatively impacts motor performance, motor skill learning rates were unaffected (Leal et al., 2024). This lack of empirical consistency highlights the need for current research approaches to better understand the consequences of SM use on motor function and skill learning.

## SM Use and Its Effect on Brain Function and Structure

Apart from SM-induced behavioral alterations on cognitive and motor function, several studies provided compelling evidence that acute SM use is associated with specific neural activation patterns in several networks such as the default mode, mentalizing, attention, and reward network (Wadsley and Ihssen, 2023). The reward network seems to be particularly sensitive to the constant stream of social rewards offered by SM platforms, such as likes and comments (Meshi et al., 2015). Both, giving and receiving likes on photo-based apps seem to activate components of the reward network such as the striatum and the ventral tegmental area in adolescents and young adults (Sherman et al., 2018). Algorithmically personalized content on video-based platforms such as TikTok has been associated with enhanced activation of the default mode network compared with nonpersonalized content (Su et al., 2021).

Apart from acute SM use effects on neural processing, long-term excessive SM use seems to affect nearly all brain networks (Hu et al., 2022) and is associated with structural alterations in gray and white matter. For example, excessive SM users show reduced gray matter volume in the ventral striatum (He et al., 2017b), the nucleus accumbens (Montag et al., 2017), and the amygdala (He et al., 2017a). Additionally, Gao et al. (2025) found that individuals with excessive short-video consumption show increased gray matter volumes in the orbitofrontal cortex and cerebellum.

Interestingly, white matter alterations as a consequence of excessive SM use have been described as well. For example, He et al. (2018) indicated interhemispheric white matter connection deficits in the corpus callosum. Furthermore, an abnormal white matter connectivity pattern in pathways that belong to reward processing and regulation, originating at the ventral striatum, was shown in excessive mobile technology users (Wilmer et al., 2019), suggesting similar alterations in excessive SM users.

SM-induced structural alterations were not only been described in young adults, but also in children. In fact, Nivins et al. (2024) found that (1) SM use did not alter the development of cortex or striatum volumes while (2) high SM use was associated with a statistically significant change in the developmental trajectory of cerebellum volumes.

Although recent studies suggest that SM use seem to affect brain function and structure, this may not necessarily be a direct consequence of frequent SM consumption. It can be speculated that also pre-existing (neurobiological) differences determine whether individuals will exhibit abnormal SM use. For example, Maza et al. (2023) provided compelling evidence that children with habitual SM checking behavior compared with nonhabitual peers showed lower baseline activity in brain regions that are involved in reward processing. Over the time course of three years, however, this activity increased in habitual checkers while it decreased in nonhabitual peers. In contrast, medium SM checking behavior was associated with no change in reward-related brain areas. Although these results suggest pre-existing differences, they indicate that the amount of SM use (checking behavior) is also capable of evoking functional brain adaptations. Nevertheless, additional longitudinal studies in different age cohorts are needed, to provide further insights into the nature/nurture debate of SM-related usage behavior.

Overall, the described structural and functional alterations associated with excessive SM use show overlaps with patterns observed in other forms of addiction (Wadsley and Ihssen, 2023). However, further research with larger sample sizes and more controlled assessments of SM use beyond predominantly self-reported measures is necessary to validate these neuroimaging findings (Wadsley and Ihssen, 2023).

## SM Use May Not Be As Bad as Previous Research Suggest

Apart from the predominantly negative effects on cognitive and motor function associated with (excessive) SM use, some research indicated that especially active SM use, defined as interacting with others or creating and engaging in SM content, may indeed have beneficial effects on physical activity and motor function. For example, López-Carril et al. (2021) provided evidence that sports students were particularly motivated and inspired to engage in physical activity through active SM use during the COVID-19 pandemic. Furthermore, active SM use has been shown to improve motivation and positive training outcomes following a 12 week workout intervention via YouTube (McDonough et al., 2022). In contrast, passive use, such as unconsciously scrolling without interaction, is often associated with detrimental effects (Thorisdottir et al., 2019).

One potential mechanism underlying these beneficial effects, beyond the physical activity itself, may be the cognitive and neural processing associated with action observation (AO). Observing others performing physical movements has been shown to activate motor brain networks, particularly the mirror neuron system, which has been shown to play a key role in imitation and motor skill learning (Rizzolatti et al., 2001). Interestingly, AO seems to recruit at least to some extend similar brain regions as compared with movement execution (Hardwick et al., 2018).

On a behavioral level, AO supports the improvement of motor abilities and motor skills (Mattar and Gribble, 2005; Gatti et al., 2019; Bazzini et al., 2023) and has been successfully implemented in both skill acquisition in sports (Kim et al., 2017) and (neuro)rehabilitation (Peng et al., 2019; Zhang et al., 2019; Ryan et al., 2021). Interestingly, the activation of the AO network seems to depend on the observer's level of expertise. Observing nonfamiliar movements show a decreased activation of the AO network in experts compared with the observation of familiar movements (Calvo-Merino et al., 2005, 2006).

AO seems to have a positive effect on cortical plasticity in the absence of physical execution. Bassolino et al. (2014) demonstrated that observing hand movements during arm immobilization prevent the typical cortico-motor suppression caused by immobilization. However, for the effectiveness of AO, the observed content should closely match the target movement (Bassolino et al., 2015) as task-specific stimuli are essential to activate the AO network.

Moreover, (selective) attention might play a crucial role for the outcome of AO since attention modulates neural responses associated with the processing of biological motion (Thompson and Parasuraman, 2012). In fact, prolonged exposure to AO has been shown to negatively impact motor skill learning outcomes (Sasaki et al., 2025). These findings highlight the need for designing AO interventions that are time-efficient, task-specific, and cognitively engaging to exploit the potential of AO in motor skill learning.

In light of the proven advantages of AO for motor skill acquisition, it is worth considering how AO principles can be applied in current digital environments such as video-based SM use. Especially, video-based SM content encourages users to observe and imitate human movements (López-Carril et al., 2021; McDonough et al., 2022). Moreover, Garcia (2025) found that students interested in fitness-related TikTok content tend to apply the observed exercises afterwards. As video-based SM content provides visual demonstrations and often includes instructional explanations, it can be valuable source for observational learning (Hussenoeder, 2022; McDonough et al., 2022). In fact, it is tempting to speculate that video-based SM content showing sports-related exercises could be used in AO-based interventions.

In contrast to AO studies in highly controlled laboratory settings, SM content potentially offers a more applicable and useful approach in everyday life situations due to the highly variable and diverse movement-specific video repertoire (Garcia, 2025).

**Figure 1.**
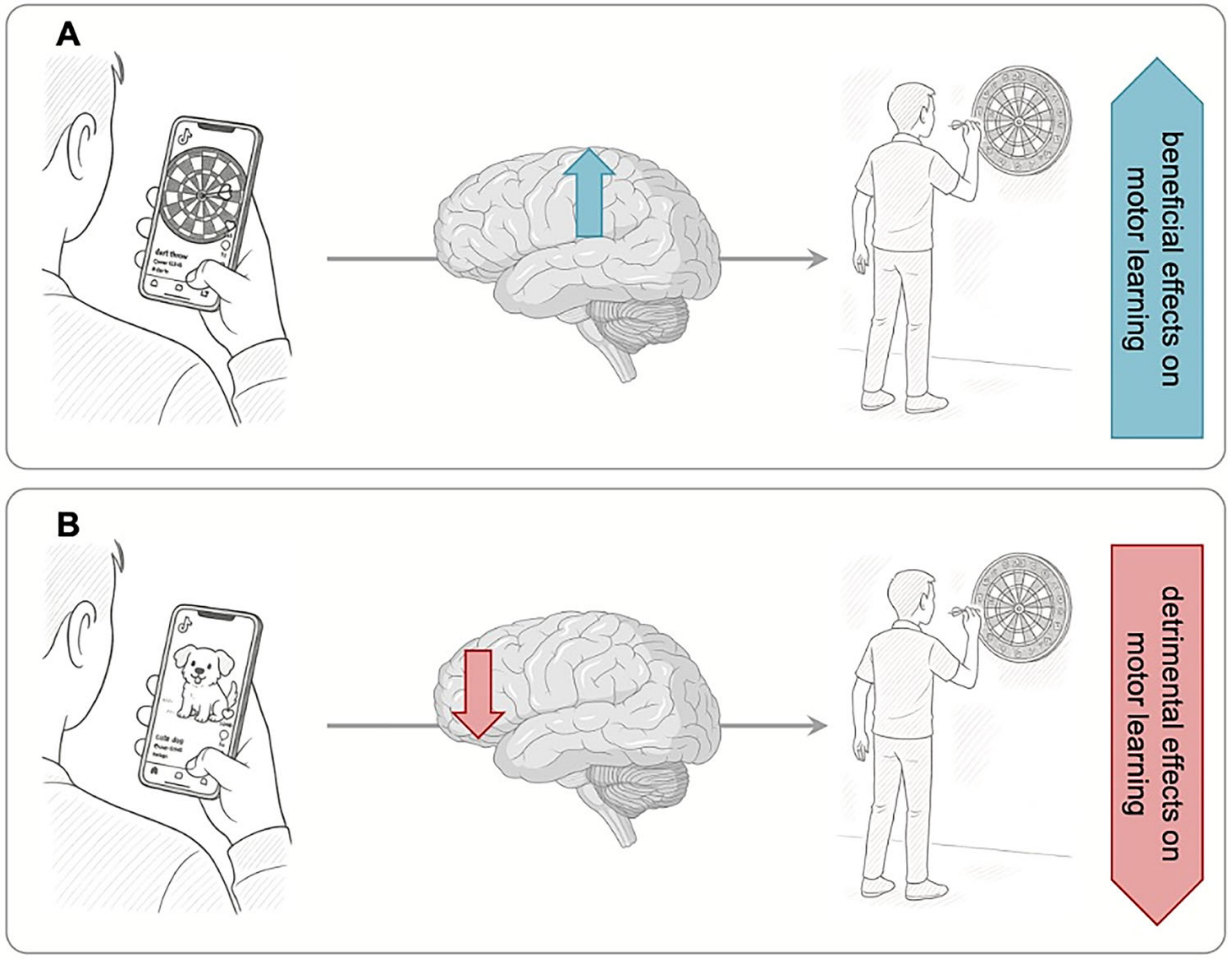
Assumed neural mechanisms underlying SM use and its effect on motor learning. ***A***, Consuming task-specific SM content may modulate functional networks (e.g., AO network) and potentially leads to beneficial effects on subsequent motor learning. ***B***, Consuming task-unspecific SM content could negatively impact neural processing and thereby could impair motor skill acquisition.

Taking this into account, it can be speculated that task-specific short-video content activates the AO network, which may be associated with a higher performance in a subsequent motor learning task (Fig. 1*A*). On the other hand, consuming task-unspecific short-video content prior to motor learning might impact attentional networks and induce mental fatigue, which, in turn, negatively impacts motor skill acquisition (Fig. 1*B*). Crucially, the proposed mechanisms remain speculative and must be validated in future research.

As SM use activates the reward system (Meshi et al., 2015), elicits strong (emotional) responses to SM content, and has platform-embedded algorithms such as endless scrolling functions through highly personalized content, users tend to remain engaged for extended periods of time (Montag et al., 2019; Goldon, 2024). Moreover, video editing features such as multi-angle perspectives and instructional explanations could be used to potentially optimize video content for motor skill learning purposes (Boucheix et al., 2018; McDonough et al., 2022). As a result, the prolonged and repeated observation of slightly different but target-specific motor actions via SM content may in fact enhance the process of motor skill acquisition.

Given the technological and motivational characteristics, we suggest that active SM use might be a promising innovative tool for educational contexts in optimizing motor skills not only in sports but also in academia (Andrew et al., 2025). Especially video-based SM platforms that provide access to active SM use can be used as educational tools that promote motivation, creativity, and engagement in a sports context and might therefore be a valuable pedagogical source for (physical) education (Escamilla-Fajardo et al., 2021; López-Carril et al., 2024; Prindle et al., 2024). Even in active SM use, the addiction risk remains and can potentially drive users into passive SM use. However, recent evidence suggest that SM in sport science or educational contexts outweigh the aforementioned risks (López-Carril et al., 2024).

Nevertheless, current guidelines have to be formulated to establish a form of digital sovereignty (Pohle and Thiel, 2020; Berkel et al., 2025). More specifically, minimum age restrictions, data privacy settings, and platform requirements have to be established to create a safe and healthy online environment. The most prominent example in this context is Australia's Online Safety Amendment, a minimum age cutoff policy of 16 years that was implemented in 2024 (Fardouly, 2025).

## Future Directions

Due to large differences in SM platforms’ characteristics, study comparisons on (negative) effects on brain and behavior remain challenging. Thus, future research should rather focus on specific SM features that can be found in most SM platforms, e.g., short-video content or giving likes. Following this, future research will be able to identify specific, feature-related neural influences that may be associated with cognition, brain function, and structure. Additionally, current research is predominantly based on self-reported SM usage behavior. Future research should focus on controlled SM assessments to provide a stronger objective outcome measure (Wadsley and Ihssen, 2023).

In general, future neuroscientific research should focus on longitudinal studies (1) to identify SM-induced influences on cognition as well as brain function and structure in more detail and (2) to understand its impact especially in different age cohorts as SM effects potentially differ relative to the users’ age. In addition, further research should clarify if pre-existing differences in brain function and structure can be a marker for excessive SM use.

Taking this into account, further research in the context of SM use and motor skill learning is needed to understand the influence of specific SM features on the acquisition of motor skills in general. As our perspective is theoretically derived but remains speculative, future research should specifically address whether and how task-specific imitation-based SM content can be incorporated into skill learning routines. In this context, it is important to clarify the optimal type of content, duration/frequency of content usage, and how these parameters are affected by age. Consistent with our perspective on future research in a general neuroscientific context, longitudinal studies are necessary for a thorough understanding of the potential beneficial effects of SM use as a playful and effective tool to augment motor skill learning.

## Synthesis

Reviewing Editor: Alexander Soutschek, Ludwig-Maximilians-Universitat Munchen Decisions are customarily a result of the Reviewing Editor and the peer reviewers coming together and discussing their recommendations until a consensus is reached. When revisions are invited, a fact-based synthesis statement explaining their decision and outlining what is needed to prepare a revision will be listed below. The following reviewer(s) agreed to reveal their identity: Samson Nivins.

The reviewers agree that the manuscript is well written and makes an interesting contribution to the current discussion of the consequences of social media use (SMU). However, the following weaknesses need to be addressed in a Revision.

Reviewers were concerned about the use of causal terms in the text. Please replace causal with correlative terms where appopriate.

It often remains unclear whether a reported study was performed in participants of young or old age. Please add age ranges for all studies, and whether a study was longitudinal or cross-sectional. Please report also effect sizes (if effect sizes were reported in a study).

Section 1, first para:

Can you add how social media is used from children to older age groups? Please add how much SMU has increased over the recent years. You may also comment on whether there is evidence for differenes between countries and female versus male users.

Moreover, a clear definition of social media is missing in the first paragraph. What do the authors consider here as social media use?

Section 2:

Can you be more explicit on whether the studies were performed on children, adolescents, or adults?

Authors state: "its long-term impact on cognitive function is still not well understood and needs to be further investigated especially over the lifespan". Yes, but can authors comment on how technology evolved over a period for e.g., 2 decades? 20 years ago media use was very different from now and it might again change within the next years, so how can long-term effects of SMU on cognition be studied?

When discussing the influence of SMU on brain development, the reviewers would appreciate if the authors could add a figure with the most relevant brain regions.

The authors should moreover consider that each developmental stage might have distinct effects of SMU.

"In fact, short-term SMU prior to physical activity has been shown to impair visuomotor performance (Fortes et al., 2022)". This is a very interesting study and worth to be explained in more detail.

Section 3:

Were these brain imaging studies based on children or adults? Please be more specific.

It is also worth discussing whether the neural changes related to SMU are a consequence of SMU or reflect pre-existing differences between social media users and non-users. The authors might also speculate on how these neural changes (e.g., in the reward system) affect behavior.

For the last para: A recent well-powered study investigated the impact of SMU on brain development longitudinally (Nivins et al., 2024, Scientific Reports). No change was seen in the cortex except for the cerebellum, which might contribute to motor skills. Please include this study in the current review.

Section 4:

The authors describe the beneficial effects of action observation (AO) on behavior, but it seems that there is no direct evidence that SMU affects the AO network. From this perspective, the assumption that SMU might have beneficial effects via AO should be taken with caution and remains speculative.

Even if (active) SMU has some positive effects, isn't there a risk that active SMU leads also to more passive SMU (as people would generally spend more time on social media)? How can we decide whether the benefits of active SMU outweigh the well-documented negative consequences?

Given the evidence for negative consequences of SMU, you may want to add a discussion of what policies should consider, e.g. minimal age cutoff or use level.

Please add an overall conclusion statement and suggest what research has to be done in the future.

## References

[B1] Alix-Fages C, González-Cano H, Baz-Valle E, Balsalobre-Fernández C (2023) Effects of mental fatigue induced by Stroop task and by social media use on resistance training performance, movement velocity, perceived exertion, and repetitions in reserve: a randomized and double-blind crossover trial. Motor Control 27:645–659. 10.1123/mc.2022-0129 37024107

[B2] Andrew K, Maj MC, Upadhya S (2025) Embracing social media trends: use of short-form videos in medical education. In: *Academic medicine - new trends and developments for the 2030s* (Stawicki SP, ed), pp 1–14. London: IntechOpen.

[B3] Bassolino M, Campanella M, Bove M, Pozzo T, Fadiga L (2014) Training the motor cortex by observing the actions of others during immobilization. Cereb Cortex 24:3268–3276. 10.1093/cercor/bht190 23897648 PMC4224244

[B4] Bassolino M, Sandini G, Pozzo T (2015) Activating the motor system through action observation: is this an efficient approach in adults and children? Dev Med Child Neurol 57:42–45. 10.1111/dmcn.12686 25690116

[B5] Bazzini MC, Nuara A, Branchini G, De Marco D, Ferrari L, Lanini MC, Paolini S, Scalona E, Avanzini P, Fabbri-Destro M (2023) The capacity of action observation to drag the trainees’ motor pattern toward the observed model. Sci Rep 13:9107. 10.1038/s41598-023-35664-w 37277395 PMC10241915

[B6] Berkel F, Bohm D, Wulff H, Reith G, Wendeborn T (2025) Digital sovereignty of physical education teachers in Germany: a situation analysis (2017–2021) on the gap between educational policies and practical implementation in further physical education teacher trainings. Front Educ 10:1544886. 10.3389/feduc.2025.1544886

[B7] Boucheix JM, Gauthier P, Fontaine JB, Jaffeux S (2018) Mixed camera viewpoints improve learning medical hand procedure from video in nurse training? Comput Human Behav 89:418–429. 10.1016/j.chb.2018.01.017

[B8] Calvo-Merino B, Glaser DE, Grèzes J, Passingham RE, Haggard P (2005) Action observation and acquired motor skills: an fMRI study with expert dancers. Cereb Cortex 15:1243–1249. 10.1093/cercor/bhi007 15616133

[B9] Calvo-Merino B, Grèzes J, Glaser DE, Passingham RE, Haggard P (2006) Seeing or doing? Influence of visual and motor familiarity in action observation. Curr Biol 16:1905–1910. 10.1016/j.cub.2006.07.065 17027486

[B10] Davis JL (2016) Social media. In: *The international encyclopedia of political communication* (Mazzoleni G, ed), Ed 1, pp 1–8. Hoboken: Wiley.

[B11] Escamilla-Fajardo P, Alguacil M, López-Carril S (2021) Incorporating TikTok in higher education: pedagogical perspectives from a corporal expression sport sciences course. J Hosp Leis Sport Tour Educ 28:100302. 10.1016/j.jhlste.2021.100302

[B12] Fardouly J (2025) Potential effects of the social media age ban in Australia for children younger than 16 years. Lancet Digit Health 7:e235–e236. 10.1016/j.landig.2025.01.016 40148007

[B13] Faro H, Franchini E, Cavalcante-Silva D, Morais da Silva RD, Barbosa BT, Gomes da Silva Machado D, Fortes LS (2025) Do prolonged social media use or cognitive tasks impair neuroelectric and visuomotor performance in taekwondo athletes? A randomized and controlled trial. Psychol Sport Exerc 76:102768. 10.1016/j.psychsport.2024.102768 39419239

[B14] Fortes LS, Berriel GP, Faro H, Freitas-Júnior CG, Peyré-Tartaruga LA (2022) Can prolongate use of social media immediately before training worsen high level male volleyball players’ visuomotor skills? Percept Mot Skills 129:1790–1803. 10.1177/00315125221123635 36028977

[B15] Gao Y, et al. (2025) Neuroanatomical and functional substrates of the short video addiction and its association with brain transcriptomic and cellular architecture. Neuroimage 307:121029. 10.1016/j.neuroimage.2025.121029 39826772

[B16] Garcia MB (2025) Watching exercise and fitness videos on TikTok for physical education: motivation, engagement, and message sensation value. J Teach Phys Educ 44:537–550. 10.1123/jtpe.2024-0084

[B17] Gatti R, Sarasso E, Pelachin M, Agosta F, Filippi M, Tettamanti A (2019) Can action observation modulate balance performance in healthy subjects? Arch Physiother 9:1. 10.1186/s40945-018-0053-0 30693101 PMC6341526

[B18] Goldon D (2024) Short videos: the real detrimental inducer for concealed brain damage. Sci Insights 44:1275–1281. 10.15354/si.24.re931

[B19] González-Padilla DA, Tortolero-Blanco L (2020) Social media influence in the COVID-19 pandemic. Int Braz J Urol 46:120–124. 10.1590/s1677-5538.ibju.2020.s121 32550706 PMC7719982

[B20] Hardwick RM, Caspers S, Eickhoff SB, Swinnen SP (2018) Neural correlates of action: comparing meta-analyses of imagery, observation, and execution. Neurosci Biobehav Rev 94:31–44. 10.1016/j.neubiorev.2018.08.003 30098990

[B21] He Q, Turel O, Bechara A (2017a) Excess social media use in normal populations is associated with amygdala-striatal but not with prefrontal morphology. Psychiatry Res Neuroimaging 269:31–35. 10.1016/j.pscychresns.2017.09.003 28918269

[B22] He Q, Turel O, Brevers D, Bechara A (2017b) Brain anatomy alterations associated with social networking site (SNS) addiction. Sci Rep 7:45064. 10.1038/srep45064 28332625 PMC5362930

[B23] He Q, Turel O, Bechara A (2018) Association of excessive social media use with abnormal white matter integrity of the corpus callosum. Psychiatry Res Neuroimaging 278:42–47. 10.1016/j.pscychresns.2018.06.008 29981941

[B24] Hu B, et al. (2022) Intersubject correlation analysis reveals the plasticity of cerebral functional connectivity in the long-term use of social media. Hum Brain Mapp 43:2262–2275. 10.1002/hbm.25786 35072320 PMC8996346

[B25] Huang C (2022) A meta-analysis of the problematic social media use and mental health. Int J Soc Psychiatry 68:12–33. 10.1177/0020764020978434 33295241

[B26] Hübner L, Voelcker-Rehage C (2017) Does physical activity benefit motor performance and learning of upper extremity tasks in older adults? A systematic review. Eur Rev Aging Phys Act 14:15. 10.1186/s11556-017-0181-7 28919929 PMC5596935

[B27] Hussenoeder FS (2022) Social network sites as learning environments and their implications for mental health. Front Digit Health 4:939740. 10.3389/fdgth.2022.939740 36300130 PMC9589159

[B28] Kemp S (2025) Digital 2026: global overview report, DataReportal – global digital insights. https://datareportal.com/reports/digital-2026-global-overview-report (October 15, 2025).

[B29] Kim T, Frank C, Schack T (2017) A systematic investigation of the effect of action observation training and motor imagery training on the development of mental representation structure and skill performance. Front Hum Neurosci 11:499. 10.3389/fnhum.2017.00499 29089881 PMC5650990

[B30] Korte M (2020) The impact of the digital revolution on human brain and behavior: where do we stand? Dialogues Clin Neurosci 22:101–111. 10.31887/DCNS.2020.22.2/mkorte 32699510 PMC7366944

[B31] Kullolli T, Trebicka B (2023) Generation Z and the evolution of social media: a two-decade analysis of impact and usage trends. Interdiscip J Res Dev 10:77. 10.56345/ijrdv10n311

[B32] Leal MG, dos Martírios Luz JE, da Silva Santos AK, Costa CL, Bandeira PF, de Miranda Meira C, Bonuzzi GM (2024) Spaced use of social media apps among motor practice trials impacts performance without influencing mental fatigue and motor learning. J Mot Learn Dev 12:333–346. 10.1123/jmld.2023-0056

[B33] López-Carril S, Escamilla-Fajardo P, Alguacil-Jiménez M (2021) Physical activity using social media during the COVID-19 pandemic: the perceptions of sports science students. Phys Cult Sport Stud Res 92:19–31. 10.2478/pcssr-2021-0022

[B34] López-Carril S, Watanabe NM, Anagnostopoulos C (2024) Tiktok as an ‘angel’ or ‘demon’ learning tool in sport sciences education: a narrative critical review. Soc Sci Humanit Open 10:101103. 10.1016/j.ssaho.2024.101103

[B35] Manwell LA, Tadros M, Ciccarelli TM, Eikelboom R (2022) Digital dementia in the internet generation: excessive screen time during brain development will increase the risk of Alzheimer’s disease and related dementias in adulthood. J Integr Neurosci 21:28. 10.31083/j.jin2101028 35164464

[B36] Martínez-González MÁ, Hu FB, Gibney MJ, Kearney J (1999) Physical inactivity, sedentary lifestyle and obesity in the European union. Int J Obes 23:1192–1201. 10.1038/sj.ijo.0801049 10578210

[B37] Mattar AAG, Gribble PL (2005) Motor learning by observing. Neuron 46:153–160. 10.1016/j.neuron.2005.02.009 15820701

[B38] Maza MT, Fox KA, Kwon SJ, Flannery JE, Lindquist KA, Prinstein MJ, Telzer EH (2023) Association of habitual checking behaviors on social media with longitudinal functional brain development. JAMA Pediatr 177:160–167. 10.1001/jamapediatrics.2022.4924 36595277 PMC9857400

[B39] McDonough DJ, Helgeson MA, Liu W, Gao Z (2022) Effects of a remote, YouTube-delivered exercise intervention on young adults’ physical activity, sedentary behavior, and sleep during the COVID-19 pandemic: randomized controlled trial. J Sport Health Sci 11:145–156. 10.1016/j.jshs.2021.07.009 34314877 PMC8487769

[B40] Meshi D, Tamir DI, Heekeren HR (2015) The emerging neuroscience of social media. Trends Cogn Sci 19:771–782. 10.1016/j.tics.2015.09.004 26578288

[B41] Montag C, et al. (2017) Facebook usage on smartphones and gray matter volume of the nucleus accumbens. Behav Brain Res 329:221–228. 10.1016/j.bbr.2017.04.035 28442353

[B42] Montag C, Lachmann B, Herrlich M, Zweig K (2019) Addictive features of social media/messenger platforms and freemium games against the background of psychological and economic theories. Int J Environ Res Public Health 16:2612. 10.3390/ijerph16142612 31340426 PMC6679162

[B43] Moreno-Llamas A, García-Mayor J, De la Cruz-Sánchez E (2020) The impact of digital technology development on sitting time across Europe. Technol Soc 63:1–6. 10.1016/j.techsoc.2020.101406

[B44] Nivins S, Sauce B, Liebherr M, Judd N, Klingberg T (2024) Long-term impact of digital media on brain development in children. Sci Rep 14:13030. 10.1038/s41598-024-63566-y 38844772 PMC11156852

[B45] O’Day EB, Heimberg RG (2021) Social media use, social anxiety, and loneliness: a systematic review. Comput Hum Behav Rep 3:100070. 10.1016/j.chbr.2021.100070

[B46] Parry DA, Fisher JT, Mieczkowski H, Sewall CJR, Davidson BI (2022) Social media and well-being: a methodological perspective. Curr Opin Psychol 45:101285. 10.1016/j.copsyc.2021.11.005 35008029 PMC9167894

[B47] Peng TH, Zhu JD, Chen CC, Tai RY, Lee CY, Hsieh YW (2019) Action observation therapy for improving arm function, walking ability, and daily activity performance after stroke: a systematic review and meta-analysis. Clin Rehabil 33:1277–1285. 10.1177/0269215519839108 30977387

[B48] Pohle J, Thiel T (2020) Digital sovereignty. Internet Policy Rev 9:1–19. 10.14763/2020.4.1532

[B49] Prindle CR, Orchanian NM, Venkataraman L, Nuckolls C (2024) Short-form videos as an emerging social media tool for STEM edutainment. J Chem Educ 101:1319–1324. 10.1021/acs.jchemed.3c01185

[B50] Ravindran T, Yeow Kuan AC, Hoe Lian DG (2014) Antecedents and effects of social network fatigue. J Assoc Inf Sci Technol 65:2306–2320. 10.1002/asi.23122

[B51] Rizzolatti G, Fogassi L, Gallese V (2001) Neurophysiological mechanisms underlying the understanding and imitation of action. Nat Rev Neurosci 2:661–670. 10.1038/35090060 11533734

[B52] Robinson LE, Stodden DF, Barnett LM, Lopes VP, Logan SW, Rodrigues LP, D'Hondt E (2015) Motor competence and its effect on positive developmental trajectories of health. Sports Med 45:1273–1284. 10.1007/s40279-015-0351-6 26201678

[B53] Ryan D, Fullen B, Rio E, Segurado R, Stokes D, O’Sullivan C (2021) Effect of action observation therapy in the rehabilitation of neurologic and musculoskeletal conditions: a systematic review. Arch Rehabil Res Clin Transl 3:100106. 10.1016/j.arrct.2021.100106 33778479 PMC7984987

[B54] Sasaki A, Suzuki E, Homma K, Mura N, Suzuki K (2025) Impact of observation duration in action observation therapy: manual dexterity, mirror neuron system activity, and subjective psychomotor effort in healthy adults. Brain Sci 15:457. 10.3390/brainsci15050457 40426628 PMC12109640

[B55] Shannon H, Bush K, Villeneuve PJ, Hellemans KG, Guimond S (2022) Problematic social media use in adolescents and young adults: systematic review and meta-analysis. JMIR Ment Health 9:e33450. 10.2196/33450 35436240 PMC9052033

[B56] Sherman LE, Greenfield PM, Hernandez LM, Dapretto M (2018) Peer influence via Instagram: effects on brain and behavior in adolescence and young adulthood. Child Dev 89:37–47. 10.1111/cdev.12838 28612930 PMC5730501

[B57] Silva FM, Duarte-Mendes P, Rusenhack MC, Furmann M, Nobre PR, Fachada MÂ, Soares CM, Teixeira A, Ferreira JP (2020) Objectively measured sedentary behavior and physical fitness in adults: a systematic review and meta-analysis. Int J Environ Res Public Health 17:8660. 10.3390/ijerph17228660 33233451 PMC7700371

[B58] Spence A, Beasley K, Gravenkemper H, Hoefler A, Ngo A, Ortiz D, Campisi J (2020) Social media use while listening to new material negatively affects short-term memory in college students. Physiol Behav 227:113172. 10.1016/j.physbeh.2020.113172 32950505

[B59] Strain T, Flaxman S, Guthold R, Semenova E, Cowan M, Riley LM, Bull FC, Stevens GA, Country Data Author Group (2024) National, regional, and global trends in insufficient physical activity among adults from 2000 to 2022: a pooled analysis of 507 population-based surveys with 5·7 million participants. Lancet Glob Health 12:e1232–e1243. 10.1016/S2214-109X(24)00150-5 38942042 PMC11254784

[B60] Su C, Zhou H, Gong L, Teng B, Geng F, Hu Y (2021) Viewing personalized video clips recommended by TikTok activates default mode network and ventral tegmental area. Neuroimage 237:118136. 10.1016/j.neuroimage.2021.118136 33951514

[B61] Thompson J, Parasuraman R (2012) Attention, biological motion, and action recognition. Neuroimage 59:4–13. 10.1016/j.neuroimage.2011.05.044 21640836

[B62] Thorisdottir IE, Sigurvinsdottir R, Asgeirsdottir BB, Allegrante JP, Sigfusdottir ID (2019) Active and passive social media use and symptoms of anxiety and depressed mood among Icelandic adolescents. Cyberpsychol Behav Soc Netw 22:535–542. 10.1089/cyber.2019.0079 31361508

[B63] Thuy D (2025) Daily time spent on social networking by internet users worldwide from 2012 to 2025, Statista. https://www.statista.com/statistics/433871/daily-social-media-usage-worldwide/ (November 19, 2025).

[B64] Wadsley M, Ihssen N (2023) A systematic review of structural and functional MRI studies investigating social networking site use. Brain Sci 13:787. 10.3390/brainsci13050787 37239257 PMC10216498

[B65] Wilmer HH, Hampton WH, Olino TM, Olson IR, Chein JM (2019) Wired to be connected? Links between mobile technology engagement, intertemporal preference and frontostriatal white matter connectivity. Soc Cogn Affect Neurosci 14:367–379. 10.1093/scan/nsz029 31086992 PMC6523422

[B66] Xu Z, Gao X, Wie J, Liu H, Zhang Y (2023) Adolescent user behaviors on short video application, cognitive functioning and academic performance. Comput Educ 203:104865. 10.1016/j.compedu.2023.104865

[B67] Zhang B, Kan L, Dong A, Zhang J, Bai Z, Xie Y, Liu Q, Peng Y (2019) The effects of action observation training on improving upper limb motor functions in people with stroke: a systematic review and meta-analysis. PLoS One 14:e0221166. 10.1371/journal.pone.0221166 31469840 PMC6716645

