# Erratum: “Is Social Media Use a Blessing or Cure for Motor Function and Skill Acquisition? An Opinion Paper”

**DOI:** 10.1523/ENEURO.0129-26.2026

**Published:** 2026-05-15

In the article “Is Social Media Use a Blessing or Cure for Motor Function and Skill Acquisition? An Opinion Paper,” by Lina Fricke, Thomas Wendeborn, and Patrick Ragert, which was published online on March 11, 2026, the title appeared incorrectly. “Cure” should instead have been “Curse.” The author regrets the error, and the online version has been corrected.

